# A review of empirical evidence on different uncanny valley hypotheses: support for perceptual mismatch as one road to the valley of eeriness

**DOI:** 10.3389/fpsyg.2015.00390

**Published:** 2015-04-10

**Authors:** Jari Kätsyri, Klaus Förger, Meeri Mäkäräinen, Tapio Takala

**Affiliations:** Department of Computer Science, School of Science, Aalto UniversityEspoo, Finland

**Keywords:** uncanny valley, human-likeness, anthropomorphism, perceptual mismatch, categorical perception, computer animation

## Abstract

The uncanny valley hypothesis, proposed already in the 1970s, suggests that almost but not fully humanlike artificial characters will trigger a profound sense of unease. This hypothesis has become widely acknowledged both in the popular media and scientific research. Surprisingly, empirical evidence for the hypothesis has remained inconsistent. In the present article, we reinterpret the original uncanny valley hypothesis and review empirical evidence for different theoretically motivated uncanny valley hypotheses. The uncanny valley could be understood as the naïve claim that any kind of human-likeness manipulation will lead to experienced negative affinity at close-to-realistic levels. More recent hypotheses have suggested that the uncanny valley would be caused by artificial–human categorization difficulty or by a perceptual mismatch between artificial and human features. Original formulation also suggested that movement would modulate the uncanny valley. The reviewed empirical literature failed to provide consistent support for the naïve uncanny valley hypothesis or the modulatory effects of movement. Results on the categorization difficulty hypothesis were still too scarce to allow drawing firm conclusions. In contrast, good support was found for the perceptual mismatch hypothesis. Taken together, the present review findings suggest that the uncanny valley exists only under specific conditions. More research is still needed to pinpoint the exact conditions under which the uncanny valley phenomenon manifests itself.

## Introduction

**Figure 1:**
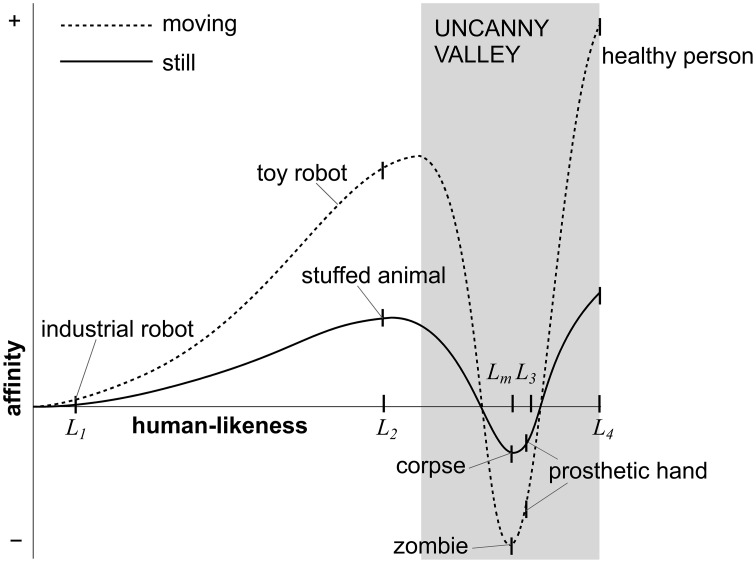
**Mori's uncanny valley curve demonstrating the non-linear relationship between the human-likeness of stimuli (clearly artificial to fully human-like) and the observers' sense of affinity for them (negative to positive)**. Human-likeness levels (*L_1_ to L4*) that correspond roughly with the turning points of the curves have been highlighted on the horizontal axis, and the uncanny valley proper has been emphasized with a dark gray color. Adapted with permission from MacDorman (2005).

Masahito Mori predicted already in the 1970s that although people would in general have favorable reactions toward increasingly humanlike robots, almost but not fully human robots would be unsettling (Mori, 2012). Mori used a hypothetical curve to characterize this relationship, and coined the sudden dip in this curve at almost humanlike levels as the uncanny valley (Figure 1). Although Mori focused on robots and other mechanical devices, the hypothesis was general enough to incorporate other domains as well. Some relevant technological innovations, such as prosthetic limbs and prototypes of anthropomorphic robots, already existed at the time when the uncanny valley hypothesis was published (cf. Mori, 2012). However, the uncanny valley hypothesis has become fully topical only during the last two decades or so, during which computer animation technologies have seen rapid advances. Although highly realistic computer-animated faces can already be produced (e.g., Alexander et al., 2010; Perry, 2014), contemporary computer animation techniques still tend to suffer from subtle imperfections related for example to rendering, lighting, surface materials, and movement dynamics. Hence, it is not surprising that the uncanny valley hypothesis has been adopted to explain the poor commercial success of some animated films in the media (cf. citations in Brenton et al., 2005; Geller, 2008; Eberle, 2009; Misselhorn, 2009; Pollick, 2010). The uncanny valley hypothesis has also motivated research in various fields beyond robotics and computer animation including, but not limited to, developmental psychology (Matsuda et al., 2012), neuroimaging (e.g., Cheetham et al., 2011; Saygin et al., 2012), animal studies (Steckenfinger and Ghazanfar, 2009), Bayesian statistics (Moore, 2012), and philosophy (Misselhorn, 2009).

Against this background, it is surprising that empirical evidence for the uncanny valley hypothesis is still ambiguous if not non-existent. Early research reviews from year 2005 noted the lack of empirical studies on the uncanny valley (Brenton et al., 2005; Gee et al., 2005; Hanson, 2005). To our knowledge, empirical evidence for the existence of the uncanny valley has still not been reviewed systematically. Several reviews have elaborated the original hypothesis and its underlying mechanisms (Ishiguro, 2006, 2007; Tondu and Bardou, 2011) or applied the original hypothesis in specific contexts (Eberle, 2009), but these reviews have not taken clear sides on the existence of the uncanny valley. Two recent reviews have concluded that the empirical evidence for the uncanny valley is either absent or inconsistent (Pollick, 2010; Zlotowski et al., 2013). These reviews have, however, cited direct evidence from relatively few studies that pertained directly to their specific fields (psychology and human-robot interaction, respectively). A possible reason for the lack of empirical research reviews could be that although a plethora of uncanny valley articles have been published, it is difficult to identify which of them have tested the original hypothesis directly and which have been merely derived from it.

It is also possible that there exist not one but many plausible uncanny valley hypotheses. Because the original uncanny valley hypothesis was intended as a broadly applicable guideline rather than an explicit experimental hypothesis (cf. Pollick, 2010), it is likely to be consistent with several more specific hypotheses. Some of these hypotheses could be derived from established psychological constructs and theories. In some cases, minor adjustments to the original uncanny valley hypothesis could be justified. Because the two major dimensions of the uncanny valley—the human-likeness of stimuli and the observers' experience of affinity for them—were not defined clearly in the original uncanny valley formulation, these dimensions could be operationalized in various different ways. Consequently, different uncanny valley studies could end up addressing different theoretical constructs and hypotheses depending on their specific methodological decisions. Because the human-likeness is difficult to operationalize, confounding factors and other alternative explanations could also limit the conclusions that can be drawn from individual studies.

The main goal of the present article was to review up-to-date empirical research evidence for a framework of plausible uncanny valley hypotheses derived from the original uncanny valley article (Mori, 2012) and other more recent publications. The review consists of five major sections. First, we will provide an interpretation of the original human-likeness and affinity dimensions of the uncanny valley (Section An Interpretation of the Uncanny Valley). We will argue that a literal interpretation of Mori's original examples, especially those involving morbid characters (i.e., corpses and zombies), would confound human-likeness with extraneous factors. We will also suggest that the original formulation of the affinity dimension could be interpreted both in terms of perceptual familiarity and emotional valence. Second, we will formulate a framework of empirically testable uncanny valley hypotheses based on the preceding analysis (Section A Framework of Uncanny Valley Hypotheses). In addition, we will reiterate the recent categorization ambiguity and perceptual mismatch hypotheses (e.g., Brenton et al., 2005; Pollick, 2010; Cheetham et al., 2011). Third, we will formulate explicit criteria for article inclusion and evaluation (Section Article Selection and Evaluation). Fourth, we will review empirical evidence for the formulated hypotheses based on the adopted evaluation criteria (Section Review of Empirical Evidence). Finally, we will discuss the implications and limitations of our findings and consider open questions in uncanny valley research (Section Discussion).

## An interpretation of the uncanny valley

### What is human-likeness?

Human-likeness is not a single quality of artificial characters that could be traced back to specific static, dynamic, or behavioral features—instead, human-likeness could be varied in an almost infinite number of different ways. Mori (2012) himself used anecdotal examples to characterize different degrees of human-likeness. We have highlighted some of these examples in Figure 1 and summarized them in Table 1. The hypothetical human-likeness levels corresponding with the selected examples have been labeled from *L*_1_ to *L*_4_. Mori used industrial robots (*L*_1_) as an example of the least humanlike characters with any resemblance to real humans. Although clearly artificial, such characters have some remotely humanlike characteristics, such as arms for gripping objects. Stuffed animals and toy robots (*L*_2_) were placed close to the first peak of the uncanny curve. Like industrial robots, these characters are clearly artificial; however, unlike industrial robots, such characters have also been purposefully designed to resemble humans. Mori placed two different kinds of objects or characters near the bottom of the valley. First, Mori mentioned prosthetic hands (*L*_3_) as an example of manmade artifacts that have been meant to appear humanlike but that have failed to do so because of some artificial qualities. Second, Mori mentioned human corpses and zombies (*L_m_*) when considering danger avoidance as a speculative explanation of the uncanny valley. Finally, Mori used healthy humans (*L*_4_) as an example of full human-likeness. In these examples, Mori referred to both static and moving instances of similar characters (e.g., still and animate corpses) to illustrate how movement would amplify the uncanny curve (Figure 1).

**Table 1:** **Focal points on the human-likeness dimension of the uncanny valley graph**.

**HL**	**Anecdotal examples**	**Human-likeness**	**Extraneous factors**	**Affinity**
*L*_1_	Industrial robot	Clearly artificial	–	Neutral
*L*_2_	Stuffed animal, toy robot	Somewhat humanlike	Aesthetics	Positive
*L*_3_	Prosthetic hand	Almost humanlike	–	Negative
*L_m_*	Corpse, zombie	Almost humanlike	Morbidity	Negative
*L*_4_	Healthy human	Fully humanlike	–	Very positive

*Anecdotal examples refer to Mori (2012)*.

*HL—degree of human-likeness*.

Table 1 also illustrates two extraneous factors that could affect affinity responses to the above anecdotal examples if they were taken literally. First, stuffed animals and toy robots could elicit positive reactions not only because they appear somewhat humanlike but because they have been purposefully designed to appear aesthetic. Similarly, human corpses, whether still or animate, would certainly not evoke negative reactions only because they appear humanlike but because they are morbid and horrifying. These considerations strongly suggest that Mori's original examples should not be adopted literally in empirical studies. However, once this approach is rejected, the question still remains which human-likeness manipulations should be used in empirical studies out of all imaginable possibilities. Although this question does not yet have an agreed upon answer, there seems to be a trend toward using image morphing and computer graphics (CG) techniques for manipulating facial stimuli in recent studies (cf. Table S1).

### What is affinity?

Mori's original Japanese terms *bukimi* and *shinwakan* (or *shin-wakan*) for the affinity dimension referred to several different concepts. The negative term *bukimi* translates quite unequivocally into eeriness (Ho and MacDorman, 2010), although other similar terms such as creepiness and strangeness have also been used (cf. Ho et al., 2008). In contrast, the positive term *shinwakan* is an unconventional Japanese word, which does not have a direct equivalent in English (Bartneck et al., 2007, 2009). The earliest and the most common translation of this term has been familiarity; however, it has been argued that likability would be a more appropriate translation (ibid.). In the latest English translation of Mori's original article, *shinwakan* was translated as affinity (Mori, 1970/2012). Similarly, we have adopted affinity when referring to the *bukimi*–*shinwakan* dimension in the present article. Table 2 lists dictionary definitions (Merriam-Webster Online Dictionary; http://www.merriam-webster.com; accessed 24.11.2014) for the most commonly used affinity terms. A closer inspection of these terms would suggest that all of them refer to various aspects of perceptual familiarity and emotional valence. Perceptual familiarity refers to recognizing that the perceived character has similar qualities as another object the observer is already well acquainted with (possibly, the observer himself or herself). Emotional valence covers various positive (liking, pleasantness, and attraction) and negative (aversive sensations) emotions elicited by the character. Although positive and negative affinity could be considered separately (e.g., Ho and MacDorman, 2010), emotional valence is an established psychological concept (e.g., Russell, 2003) that is able to incorporate both of them.

**Table 2:** **Dictionary definitions for the common English translations of Mori's affinity dimension**.

**English term**	**Definitions**
Eeriness	[The quality of being] strange and mysterious
	[…] so mysterious, strange, or unexpected as to send a chill up the spine
Likability	[…] easy to like
	[…] pleasant or appealing
	[… bringing] about a favorable regard
Familiarity	The state of being [well acquainted] with something
	[…] having knowledge about something
	A state of close relationship [similar to intimacy]
Affinity	A feeling of closeness and understanding that someone has for another person because of their similar qualities, ideas, or interests
	A liking for or an attraction to something
	The state of being similar or the same

Given that the original terms for the affinity dimension (or at least their common translations) are ambiguous, empirical studies would be necessary for resolving which self-report items would be ideal for measuring affinity. Previous studies have suggested that eeriness is associated with other negative emotion terms such as fear, disgust, and nervousness (Ho et al., 2008); or fear, unattractiveness, and disgust (Burleigh et al., 2013). To our knowledge, only one previous study up to date has used factor analytic methods to develop a conclusive self-report questionnaire for uncanny valley studies (Ho and MacDorman, 2010). This study identified orthogonal factors for human-likeness, eeriness (two separate factors: eerie and spine-tingling), and attractiveness. An informal evaluation would suggest some potential problems with this questionnaire, however. First, some of the questionnaire items are not necessarily ideal for measuring their intended constructs in all contexts. For example, the semantic differential items “ordinary—supernatural” and “without definite lifespan—mortal” could be inappropriate human-likeness measures when none of the evaluated stimuli are supernatural. Second, although the identified eeriness factors are consistent with Mori's original terms, their constituent items (e.g., “numbing—freaky” and “unemotional—hair-rising”) do not resemble items in typical emotion self-report questionnaires (cf. self-report items in Bradley and Lang, 1994). Third, familiarity items were not considered in the study, although familiarity would seem to be an integral part of the uncanny valley. Although future empirical studies might be useful for refining this scale, this work is an important step toward developing a common metric for the affinity dimension. The scale has already been applied in at least two studies (Mitchell et al., 2011; MacDorman et al., 2013).

## A framework of uncanny valley hypotheses

**Figure 2:**
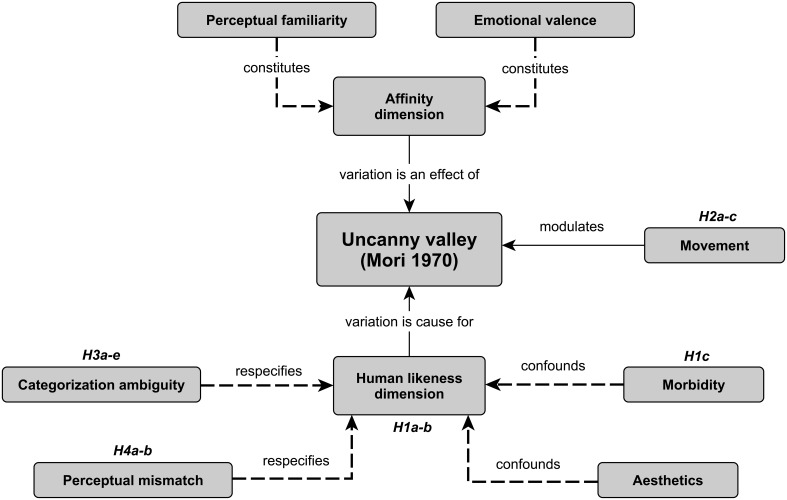
**A concept map demonstrating relations between the present uncanny valley hypotheses and different uncanny valley concepts derived from Mori (2012)**. Dashed lines refer to constructs that have been explicated after Mori's original publication. Hypotheses: H1a—naïve UV proper, H1b—naïve HL, H1c—morbidity, H2a—UV proper for movement, H2b—HL for movement, H2c—movement modulation, H3a—category identification, H3b—perceptual discrimination, H3c—categorical identification difficulty, H3d—opposite perceptual discrimination, H3e—perceptual discrimination difficulty, H4a—inconsistent HL, H4b—atypicality; UV—uncanny valley, HL—human-likeness.

Figure 2 illustrates the preceding analysis of the uncanny valley phenomenon (Section An Interpretation of the Uncanny Valley) and the relations between the present hypotheses and the uncanny valley concepts.

### Naïve hypotheses

**Figure 3:**
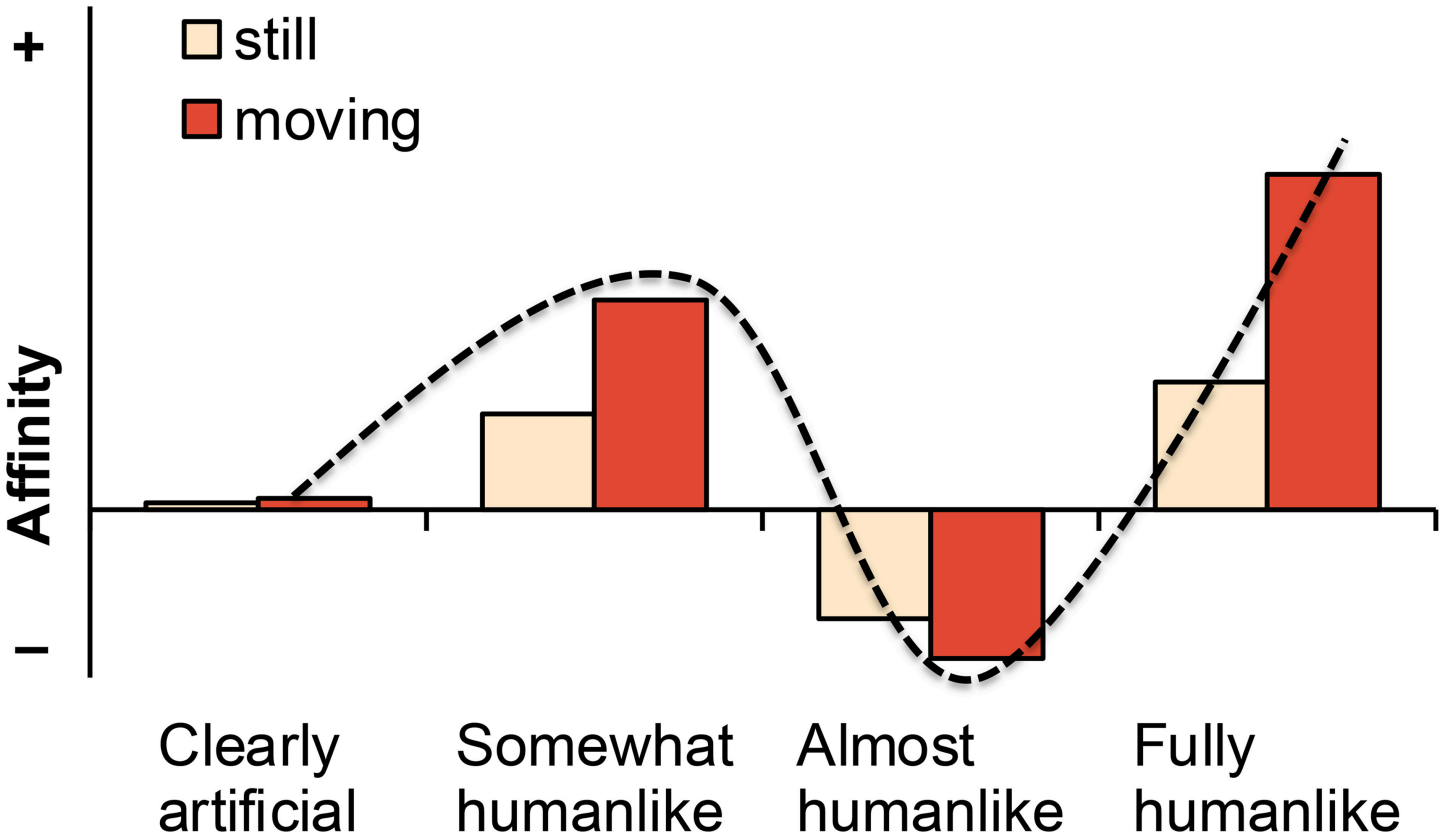
**Predicted affinity levels (from negative to positive) for still and moving versions of characters representing different human-likeness levels**. The characteristic uncanny curve is overlain on the data for illustration.

The question of which specific human-likeness manipulations should be used in empirical uncanny valley studies could be sidestepped by assuming that *any* kind of manipulation would lead to the characteristic uncanny curve for affinity (Figure 1). However, this hypothesis is simplistic because it assumes that all imaginable human-likeness manipulations are equally relevant for the uncanny valley. Consequently, it could be referred to as a naïve uncanny valley hypothesis as opposed to more specific hypotheses (Section Refined Hypotheses). We have attempted to formulate this hypothesis so that it would be compatible with various human-likeness manipulations ranging from categorical manipulations with a minimal number of human-likeness levels to fully continuous manipulations. Figure 3 illustrates the original uncanny curve for the four most focal human-likeness levels (Table 1). These levels constitute the minimal set of human-likeness levels that could be used to capture the most relevant aspects of the original uncanny curve.

The core claim of the uncanny valley is that almost humanlike characters will elicit more negative affinity than any other characters (Figure 3). As can be seen in the darkened region of Figure 1, this characteristic U-shaped curve forms the uncanny valley proper. Because almost humanlike characters would need to be compared to both more artificial and more humanlike characters, the bare minimum for testing this prediction would be three human-likeness levels (cf. Figure 3). Although not equally critical, the original uncanny valley hypothesis also predicts that, except for the uncanny valley proper, affinity will be more positive for increasingly humanlike characters. That is, affinity increases when moving from clearly artificial to somewhat humanlike characters, and there would also be a relative increase between somewhat and fully humanlike characters (Figure 3). Given that this hypothesis omits almost humanlike characters, at least the remaining three levels in Figure 3 would need to be used to test this prediction. These predictions can be formulated as the following hypotheses.

*H1a (“naïve uncanny valley proper”): For any kind of human-likeness manipulation, almost humanlike characters will elicit more negative affinity (lower familiarity and/or more negative emotional valence) than any other more artificial or more humanlike characters*.

*H1b (“naïve human-likeness”): For any kind of human-likeness manipulation, more humanlike characters will elicit more positive affinity (higher familiarity and/or more positive emotional valence), with the possible exception of characters fulfilling H1a*.

### Morbidity hypothesis

Although purposefully morbid characters could be adopted from the original uncanny valley formulation (Mori, 2012) and used in empirical uncanny valley studies, such characters would confound the more interesting effects of varying human-likeness (Section What is Human-Likeness?). Although it is quite trivial that such characters should evoke negative affinity, we have nevertheless formulated the following hypothesis to help separate morbidity effects from those of other hypotheses.

*H1c (“morbidity”): Morbid characters (e.g., corpses or zombies) will elicit more negative affinity (lower familiarity and/or more negative emotional valence) than any other characters*.

### Movement hypotheses

In his original formulation, Mori (2012) also suggested that movement would amplify the uncanny curve. That is, the positive and negative affinity experiences elicited by the still characters should become more pronounced for moving characters. The role of movement could, however, be more complex than originally predicted. For example, although Mori considered movement as a dichotomous variable—it either is or is not present—movement features could also range in human-likeness and lead to an uncanny curve of their own. This leads to the following reformulations of the naïve uncanny hypotheses (H1a and H1b).

*H2a (“uncanny valley proper for movement”): For any kind of human-likeness manipulation, “almost humanlike” movement patterns will elicit more negative affinity (lower familiarity and/or more negative emotional valence) than any other more artificial or more humanlike movement patterns*.

H2b (“human-likeness for movement”): For any kind of human-likeness manipulation, more humanlike movement patterns will elicit more positive affinity (higher familiarity and/or more positive emotional valence), with the possible exception of movement patterns fulfilling H2a

The original movement hypothesis can be stated as follows.

*H2c (“movement modulation”): Movement will amplify the affinity responses (changes in familiarity and/or emotional valence) associated with hypotheses H1a and H1b*.

Testing movement hypotheses H2a–c would require the same number of minimum human-likeness levels as the more general hypotheses H1a and H1b (that is, three levels; the specific levels depending on the hypothesis).

### Refined hypotheses

#### Categorization ambiguity

Early uncanny valley postulations have suggested that negative affinity would be caused by the ambiguity in categorizing highly realistic artificial characters as real humans or artificial entities (e.g., Ramey, 2005 [quoted in MacDorman and Ishiguro, 2006]; Pollick, 2010). Notably, this suggestion itself does not yet consider whether the human-likeness dimension of the uncanny valley is perceived continuously or categorically—that is, some intermediate characters could be difficult to categorize regardless of whether increasing human-likeness were perceived as a gradual continuum or discretely as artificial and human categories.

Categorical perception, which is an empirically and theoretically established construct in psychology, has been applied to the uncanny valley in recent empirical studies (Cheetham et al., 2011, 2014). Loosely speaking, categorical perception refers to the phenomenon where the categories possessed by an observer influence his or her perceptions (Goldstone and Hendrickson, 2010). Specifically, categorical perception is thought to occur when the perceptual discrimination is enhanced for pairs of perceptually adjacent stimuli straddling a hypothetical category boundary between two categories, and decreased for equally spaced pairs belonging to the same category (Repp, 1984; Harnad, 1987; Goldstone and Hendrickson, 2010). Applied to the uncanny valley, categorical perception would mean that “[… ] irrespective of physical differences in humanlike appearance, objects along the DOH [degree of human-likeness] are treated as conceptually equivalent members of either the category ‘non-human’ or the category ‘human,’ except at those levels of physical realism at the boundary between these two categories.” (Cheetham et al., 2011, p. 2).

The two most commonly agreed upon criteria for experimental demonstrations of categorical perception are category identification and perceptual discrimination (Repp, 1984; Harnad, 1987). The identification criterion means that stimulus identification in a labeling task should follow a steep slope such that labeling probabilities change abruptly at the hypothetical category boundary. Given that the location of category boundary cannot be known in advance, the minimum number of required stimulus levels for testing this hypothesis cannot be determined precisely. In practice, previous uncanny valley studies have employed at least 11 evenly distributed human-likeness steps along the human-likeness continuum (e.g., Looser and Wheatley, 2010; Cheetham et al., 2011, 2014). Response times have been used as an index of uncertainty in the identification task (e.g., Pisoni and Tash, 1974; de Gelder et al., 1997). Assuming that categorization ambiguity should be the greatest at the category boundary, the slowest response times should also coincide with this point. That is,

*H3a (“category identification”): A steep category boundary will exist on the human-likeness axis such that characters on the left and right sides of this boundary are labeled consistently as “artificial” and “human,” respectively; and/or this identification task will elicit the slowest response times at the category boundary*.

The discrimination criterion refers to the above requirement that perceptual discrimination should be better for stimulus pairs straddling the category boundary than for equally spaced stimulus pairs falling on the same side of the category boundary. As an example from previous work (Cheetham et al., 2011), the four stimulus pairs artificial–artificial, artificial–human, human–artificial, and human–human could be derived from identification results and employed in the perceptual discrimination task. All possible stimulus pairs that are differentiated by an equal number of steps on the human-likeness continuum could also be used (e.g., Cheetham et al., 2014). As a summary, to demonstrate categorical perception for the human-likeness dimension of the uncanny valley, the following hypothesis should be confirmed in addition H3a.

*H3b (“perceptual discrimination”): Character pairs that straddle the category boundary between “artificial” and “human” categories will be easier to discriminate perceptually than equally different character pairs located on the same side of the boundary*.

After demonstrating that the human-likeness dimension is perceived categorically, it would still need to be shown that category identification difficulty (i.e., H3a) is also associated with subjective experiences of negative affinity. The most straightforward assumption would be that identification uncertainty at the category boundary (“categorization ambiguity”) leads to negative affinity. Strictly speaking, this hypothesis is not fully consistent with categorical perception as it is commonly understood, given that the hypothesis refers only to the category identification criterion (cf. H3a), whereas perceptual discrimination criterion (H3b) has been considered as the hallmark of categorical perception (e.g., Harnad, 1987). Hence, categorization ambiguity could lead to negative affinity even in the absence of categorical perception (i.e., when only H3a but not H3b holds true). However, we have included this hypothesis, as it is consistent with the early uncanny valley literature (Ramey, 2005 [quoted in MacDorman and Ishiguro, 2006]; Pollick, 2010). Hence, we have formulated this hypothesis as follows.

*H3c (“categorical identification difficulty”): Characters that are located at the category boundary between “artificial” and “human” categories (as identified in H3a-b) will elicit more negative affinity (lower familiarity and/or more negative emotional valence) than any other characters that are located on the left or right sides of the category boundary*.

As suggested recently by Cheetham et al. (2014), the original uncanny valley hypothesis is based on the implicit assumption that perceptual discrimination is the most difficult for characters in the uncanny valley. However, assuming that the uncanny valley proper is thought of as coinciding with the category boundary and that this boundary is considered in terms of the categorical perception framework, it follows (as in Cheetham et al., 2014) that perceptual discrimination performance should actually be easier for characters at or in the close vicinity of the category boundary and not more difficult. As can be seen, this is the position taken in hypothesis H3b, and the perceptual discrimination difficulty assumption would be its opposite. Assuming that perceptual discrimination would be more difficult for characters in the uncanny valley, this difficulty should also be associated with negative affinity. These hypotheses can be stated as follows.

*H3d (“opposite perceptual discrimination”): Character pairs that straddle the category boundary between “artificial” and “human” categories will be more difficult to discriminate perceptually than equally different character pairs located on the same side of the boundary*.

*H3e (“perceptual discrimination difficulty”): Increased perceptual discrimination difficulty for adjacent character pairs will be associated with heightened negative affinity (lower familiarity and/or more negative emotional valence)*.

#### Perceptual mismatch

Hypotheses H3a-e are attractive because they are related to the well-established framework of categorical perception. However, there are several reasons for considering also other alternatives to these categorization ambiguity and categorical perception based explanations. First, there is no *a priori* reason for expecting that the human-likeness dimension should be perceived categorically rather than continuously. For example, Campbell et al. (1997) has demonstrated that whereas morphed continua between human and cow faces are perceived categorically, similar continua between humans and monkeys are continuous. Similarly as humans and other primates, humans and anthropomorphic characters share many fundamental similarities that could place them in the same overarching category of humanlike entities (cf. Campbell et al., 1997; Cheetham et al., 2011). Second, negative affinity could of course be caused by some other mechanisms in addition to (or instead of) categorization ambiguity or categorical perception. For example, it is conceivable that some characters on the “human” side of the category boundary would be considered eerie because they appeared human but contained features that are not “entirely right.” In this hypothetical but conceivable example, a negative affinity peak would be located on the right side of the category boundary.

The perceptual mismatch hypothesis, which is theoretically independent from the categorization ambiguity and categorical perception hypotheses, has been presented recently as another explanation for the uncanny valley (e.g., MacDorman et al., 2009; Pollick, 2010). This hypothesis suggests that negative affinity would be caused by an inconsistency between the human-likeness levels of specific sensory cues. Clearly artificial eyes on an otherwise fully human-like face—or vice versa—is an example of such inconsistency. A particularly interesting proposal is that negative affinity would be caused by inconsistent static and dynamic information (Brenton et al., 2005; Pollick, 2010). The bare minimum for testing this hypothesis would be four experimental manipulation levels (i.e., two realism levels × two different features). We have formulated this hypothesis in more general terms below.

*H4a (“inconsistent human-likeness”): Characters with inconsistent artificial and humanlike features will elicit more negative affinity (lower familiarity and/or more negative emotional valence) than characters with consistently artificial or characters with consistently humanlike features*.

Another form of perceptual mismatch could be higher sensitivity to deviations from typical human norms for more humanlike characters (e.g., Brenton et al., 2005; MacDorman et al., 2009). Deviations from human norms could result, for example, from such atypical features as grossly enlarged eyes. In the uncanny valley context, a plausible explanation for this phenomenon could be that the human visual system has acquired more expertise with the featural restrictions of other humans than with the featural restrictions of artificial characters (cf. Seyama and Nagayama, 2007). This hypothesis is also consistent with previous studies demonstrating that faces with typical or average features are considered more attractive than atypical faces (e.g., Langlois and Roggman, 1990; Rhodes et al., 2001). The atypicality hypothesis is similar to the above inconsistency hypothesis, given that atypical features could also be considered artificial. In fact, these two hypotheses have previously been considered as the same hypothesis (e.g., MacDorman et al., 2009). However, the atypicality hypothesis could refer to any deviant features besides artificiality (e.g., any distorted human features) and, unlike the inconsistency hypothesis, it makes a unilateral prediction related to only humanlike characters. Testing atypicality would require at least four experimental manipulation levels (artificial without atypical features, artificial with atypical features, human without atypical features, human with atypical features), and it could be formulated as follows.

*H4b (“atypicality”): Humanlike characters with atypical features will elicit more negative affinity (lower familiarity and/or more negative valence) than artificial characters with atypical features, or either humanlike or artificial characters without atypical features*.

#### Relation to the original uncanny valley hypothesis

The above hypotheses can be seen as refinements of the original uncanny valley hypothesis such that each of them narrows the human-likeness conditions under which the uncanny valley is expected to occur. These hypotheses pertain only to the uncanny valley proper (i.e., the “almost humanlike” level), and they cannot account for the first peak in the uncanny curve (cf. H1b and Figure 3). Otherwise, all of these hypotheses would appear to be consistent with the original uncanny valley hypothesis. For example, all of them seem to be consistent with the following quote: “One might say that the prosthetic hand has achieved a degree of resemblance to the human form [… ]. However, once we realize that the hand that looked real at first sight is actually artificial, we experience an eerie sensation.” (Mori, 2012, p. 99; see also MacDorman et al., 2009, p. 698). Here, the prosthetic hand could have appeared eerie because it caused an artificial–human category conflict (H3), it was perceived as containing mismatching artificial and human features (H4a), or because the hand resembled a real hand without fulfilling all of the typical characteristics of human hands (H4b).

## Article selection and evaluation

### Evaluation criteria

Table 3 displays the criteria that were used for selecting individual studies and for evaluating their results. These criteria are based on the general validity typology of Shadish et al. (2002), which describes four different types of validity and their associated threats. Our goal was to identify justifiable and plausible threats for conclusions that can be drawn from the reviewed studies to hypotheses H1–H4. Hence, we have not attempted to develop a comprehensive list of all possible threats to the experimental validity of individual studies.

**Table 3:** **Evaluation criteria for possible threats that limit the conclusions that could be drawn from individual studies to the present hypotheses**.

**Threat**	**Validity type**
No or inadequate statistical tests^a^	Statistical conclusion
Heterogeneous stimuli	Statistical conclusion
No manipulation check for human-likeness^a^	Internal
Image morphing artifacts	Internal
Categorical perception not tested^b^	Construct
Irrelevant affinity measures^a^	Construct
Familiarity evaluations misunderstood	Construct
Outlier stimuli (e.g., morbid characters)	Construct
Alternative explanations	Construct
Narrow human-likeness range	Construct
Narrow set of manipulated stimuli	Construct
Narrow participant sample	External

*Validity types refer to Shadish et al. (2002)*.

a *Used as article inclusion criteria*.

b *Applies only to the hypotheses H3c and H3e*.

#### Statistical conclusion validity

Statistical conclusion validity refers to the validity of inferring that the experimental manipulations and measured outcomes covaried with each other. At the bare minimum, any kind of statistical test should be used to provide evidence against chance results. The predicted U-shaped relationship between human-likeness and affinity (Figure 1) could be tested, for example, by using second-order correlation tests or analysis of variance followed by *post-hoc* comparisons. Linear correlation test would, however, not be sufficient for testing the predicted nonlinear relationship. Statistical conclusion validity could also be compromised by uncontrolled variation in the stimuli. This issue could be a particular concern for realistic stimuli (e.g., video game characters), whose features cannot be fully controlled. Extraneous variation could possibly be reduced by careful pretesting of stimuli and the inclusion of a large number of stimuli for each stimulus category.

#### Internal validity

Internal validity refers to whether the observed outcomes were caused solely by experimental manipulations or whether they would have occurred even without them. Failure to check or confirm that human-likeness manipulations elicited consistent changes in perceived human-likeness would raise doubts over whether human-likeness was actually varied as intended, and would hence threaten internal validity.

Artifacts produced by human-likeness manipulations could also be considered as threats to internal validity (strictly speaking, these and any other confounds would be threats to construct validity in the original typology; cf. Shadish et al., 2002, p. 95). We will consider image morphing artifacts in detail because this method has become popular in uncanny valley studies (cf. Table S1). Image morphing procedure is used to construct a sequence of gradual changes between two images (e.g., CG and human faces), and it consists of three phases: geometric correspondence is established between the images, a warping algorithm is applied to match the shapes of the original objects, and color values are interpolated between the original and warped images (e.g., Wolberg, 1998). Image morphing algorithms are prone to at least two kinds of artifacts (e.g., Wu and Liu, 2013). First, ghosting or double-exposure between images can occur if they contain different features, geometric correspondence has not been established adequately, or warping has not been applied. Second, color interpolation typically causes some blurring because it combines values from several pixels in the original images. Image morphing artifacts are a threat to validity because they are likely to coincide with intermediate levels of human-likeness (i.e., the most processed images). Cheetham and Jäncke (2013) have published a detailed guideline for applying morphing to facial images in uncanny valley studies. We have adopted the following criteria from their guideline: (i) several morphed continua should be used, (ii) selected endpoint images should be similar to each other (i.e., the faces should have similar geometries, have neutral facial expressions, and represent individuals of similar ages), (iii) alignment disparities should be avoided, and (iv) any external features should be masked (i.e., hair and ears, jewelry, and other external features).

#### Construct validity

Construct validity refers to the extent to which the experimental manipulations and measured outcomes reflect their intended cause and effect constructs. For example, if the categorization ambiguity hypothesis (H3c or H3e) were demonstrated for specific stimuli without also demonstrating that these stimuli indeed were perceived categorically (H3a–b), it could be uncertain whether categorical perception was in fact involved. For the present purposes, we have required that the outcome measures should tap into the perceptual familiarity and/or emotional valence constructs (Section What is Affinity?). A specific threat related to self-reported familiarity is that it could be confounded with previous experience (e.g., a video game character could be familiar because of its popularity). The inclusion of outlier stimuli that represent other constructs besides varying human-likeness, for example morbidity (Section What is Human-Likeness?), would also threaten construct validity. In the present context, the hypothesis H1c was intended to set such constructs apart from human-likeness. It is also possible that affinity changes could in some cases be explained by other alternative constructs or phenomena (e.g., poor lip synchronization). A narrow range of manipulated human-likeness (e.g., only CG characters) could threaten construct validity because the results would not necessarily generalize to the full range of human-likeness. Application of human-likeness manipulations to only a single stimulus character could also threaten construct validity, if it were plausible that the manipulation results would contain other irrelevant features in addition to or instead of human-likeness.

#### External validity

External validity refers to what extent the observed causal relationship between manipulated and observed variables can be generalized to other participants, experimental manipulations, and measured outcomes. Generalizability could be considered by comparing results from different studies. In practice, this would be difficult because of the heterogeneity of uncanny valley studies (cf. Table S1). For the present purposes, we have considered external validity only to exclude results from individual studies with clearly unrepresentative participant samples (e.g., only children).

### Article selection

We identified empirical uncanny valley studies by searching for the key term “uncanny valley” in the following search engines: Scopus (search in article title, abstract, and keywords; including secondary documents; *N* = 273), PubMed (search in all fields; *N* = 23), Science Direct (search in all fields; *N* = 134), and Web of Science (search in topic; *N* = 114). The obtained list of articles was augmented by other articles cited in them and by articles identified from other sources (*N* = 6). This initial list (*N* = 550) was screened by the first author. Duplicate entries and other than full-length articles published in peer-reviewed journals or conference proceedings were removed semi-automatically, and a cursory selection was done to exclude studies that had clearly not tested or considered the present hypotheses.

The screened list (*N* = 125) was evaluated by all authors for eligibility. The following inclusion criteria were used (cf. Table 3): (i) the study had addressed, implicitly or explicitly, at least one of the hypotheses H1–H4; (ii) the study had used at least the minimum number of human-likeness levels for each hypothesis (cf. Section A Framework of Uncanny Valley Hypotheses); (iii) human-likeness of stimuli had been tested explicitly and confirmed; (iv) unless irrelevant for the tested hypothesis (i.e., H3a, H3b, and H3d), the study had used any of the conventional self-report items (likability, eeriness, familiarity, or affinity) or their equivalents for measuring affinity responses; and (v) justified statistical test had been used for testing the relationship between human-likeness and affinity. Two studies that had not tested human-likeness explicitly (Seyama and Nagayama, 2007; Mäkäräinen et al., 2014) were nevertheless included because their human-likeness manipulations (image morphing from artificial to human faces and increasingly more abstract image manipulations, respectively) should have been expected to elicit trivial changes in perceived human-likeness. The final list of selected articles (*N* = 17) is given in Table S1.

### Article and hypothesis evaluation

The validity of conclusions from individual studies to hypotheses H1–H4 was evaluated using those evaluation criteria in Table 3 that had not already been adopted as inclusion criteria. All threats that were considered possible are listed in Table S1; however, only those threats that were considered both plausible and relevant for a specific hypothesis were used for excluding individual results. To allow critical evaluation and possible reanalysis of the present findings, we have attempted to highlight potential controversies related to the inclusion and evaluation of studies when reviewing the evidence for each hypothesis.

Because the selected articles had used heterogeneous methodologies and most of them had not reported effect size statistics, a quantitative meta-analysis would not have been appropriate. Instead, we opted to present the numbers of findings providing significant and non-significant evidence for each hypothesis. Because significant findings opposite to hypotheses were rare, they were pooled with the non-significant findings. Significant opposite findings have been mentioned separately in the text. Although this kind of “box score” approach is inferior to quantitative meta-analytic methods (Green and Hall, 1984), it can nevertheless be used to provide an overall quantification of result patterns in the reviewed literature. Following a previous recommendation (Green and Hall, 1984), we adopted a 30% threshold for deciding how many positive findings would be considered significant evidence in favor of a specific hypothesis. All of the reported findings were clearly above this threshold.

## Review of empirical evidence

### Naïve and morbidity hypotheses

Empirical evidence for naïve, morbidity, and movement hypotheses is presented in Table 4. Whereas the results clearly confirmed that affinity increased linearly across increasing human-likeness (H1b; 7 out of 9 studies), the predicted uncanny valley proper (H1a) received almost no support (1 out of 8 studies). As an exception, one study showed that pictures of intermediate prosthetic hands were more eerie than pictures of either mechanical or human hands (Poliakoff et al., 2013). Two other studies provided results that resembled the uncanny curve (McDonnell et al., 2012; Piwek et al., 2014); however, closer inspection suggested that these results could have been explained by outlier stimuli—that is, in terms of the hypothesis H1c. Another one of these studies (McDonnell et al., 2012) could have provided evidence for H1a even after the outlier stimulus (purposefully ill character) was excluded. However, we considered this evidence inconsistent because both unrealistic (“ToonBare” rendering) and realistic (“HumanBasic” rendering) stimuli were found to be less appealing, friendly, and trustworthy than the remaining stimuli.

**Table 4:** **Empirical evidence for hypotheses H1 (naïve hypotheses and morbidity) and H2 (movement)**.

**Author/year**	**H1a**	**H1b**	**H1c**	**H2a**	**H2b**	**H2c**
Seyama and Nagayama, 2007	−	−				
MacDorman et al., 2009	−	+				
Looser and Wheatley, 2010	−	+				
Thompson et al., 2011				−	+	
McDonnell et al., 2012	(+)	+	+			(+)
Yamada et al., 2013	(+)	(−)				
Burleigh et al., 2013	−	+				
Carter et al., 2013	−	+				
Poliakoff et al., 2013	+	(+)				
Cheetham et al., 2014	−	+				
Piwek et al., 2014	(+)	−	+	−	+	(−)
Rosenthal—von der Pütten and Krämer, 2014	−	+				
Total	8	9	2	2	2	0
+	1	7	2	0	2	0
−	7	2	0	2	0	0

*Conclusions: “+”: significant in favor of the hypothesis, and “−”: non-significant or significant against the hypothesis. Conclusions in parentheses have been omitted from total scores because of plausible threats to validity. Hypotheses: H1a—naïve UV proper, H1b—naïve HL, H1c—morbidity, H2a—UV proper for movement, H2b—HL for movement, H2c—movement modulation. UV—uncanny valley, HL—human-likeness*.

One of the studies in Table 4 (Yamada et al., 2013) was excluded from the total count because of plausible morphing artifacts. This study found a U-shaped curve for self-reported pleasantness vs. morphed human-likeness, which could have been taken as support for H1a. However, in this study, only one pair of images had been selected for creating the human-likeness continuum, the selected cartoon and human face were very dissimilar from each other, and no masking had been used (cf. Section Article Selection; and Cheetham and Jäncke, 2013). Hence, it is possible that the lower pleasantness ratings for intermediate morphs could have resulted from morphing artifacts rather than intermediate human-likeness level. Consistently with this interpretation, other morphing studies (Looser and Wheatley, 2010; Cheetham et al., 2014) with masked faces and multiple matched face pairs have failed to find a similar U-shaped curve for participants' evaluations. Another morphing study in Table 4 (Seyama and Nagayama, 2007) had also used unmasked and quite dissimilar face pairs; however, it is unlikely that the lack of significant findings in this study could have been explained by morphing artifacts.

Several other potentially interesting studies were excluded during the initial selection and were hence not included in Table 4 or Table S1. For example, seminal uncanny valley studies (Hanson, 2006; MacDorman, 2006; MacDorman and Ishiguro, 2006) were excluded because these studies did not report statistical test results for their findings. Because these studies also seemed to be influenced by morphing artifacts or the use of heterogeneous stimuli, their results for hypotheses H1a–b would nevertheless have been excluded as per our evaluation criteria. Results from several studies using realistic video game (or similar) characters have also been excluded either because they had not used statistical tests or because they had tested only linear correlations statistically. Most of the excluded studies had also deliberately included outlier characters (e.g., zombies) in their experimental stimuli (e.g., Schneider et al., 2007; Tinwell et al., 2010) and some of their results could have been explained by alternative explanations (e.g., audiovisual asynchrony; Tinwell et al., 2010, in press). We were able to identify only one published study without such outlier characters (Flach et al., 2012) that could be taken as tentative evidence for H1a. This study demonstrated an uncanny curve for experienced discomfort (measured as a dichotomous variable) across video game and film characters that represented different human-likeness levels. We considered this evidence tentative because no statistical tests had been used; furthermore, the human-likeness range was somewhat constrained by the use of only CG characters.

### Movement hypotheses

We were able to identify only two studies (Thompson et al., 2011; Piwek et al., 2014) that could be taken as evidence for the independent movement hypotheses H2a and H2b (Table 4). Results from these two studies were, however, consistent with those of the more general hypotheses H1a–b. That is, more humanlike movement was found to elicit higher affinity (H2b) in both studies, whereas a nonlinear uncanny valley curve (H2a) was not observed in either one of them. No studies addressing the modulatory effect of movement (H2c) survived the initial selection and further evaluation. Two studies demonstrated modulatory movement effects; however, these effects were specific to plausible outlier characters (ill-looking face in McDonnell et al., 2012; and zombie character in Piwek et al., 2014). Furthermore, these studies provided conflicting evidence: the former reported a significant increase and the latter a significant decrease in negative affinity for the moving characters.

### Categorization ambiguity hypotheses

Empirical evidence for categorization ambiguity (H3) and perceptual mismatch (H4) hypotheses is presented in Table 5. Four studies demonstrated that a category boundary existed for the identification of morphed facial image continua (H3a) and three of these studies additionally demonstrated that discrimination performance reached its peak when the images straddled this category boundary (H3b). The opposite prediction that discrimination performance would be the poorest in the vicinity of category boundary (H3d) was not supported by any study. These results hence provided reasonable evidence for the categorical perception of morphed human-likeness continua. In contrast, we managed to identify only two studies that tested affinity responses elicited by categorization ambiguity (H3c); neither of which could be taken as evidence in favor of this hypothesis. Opposite to hypothesis H3e, one study (Cheetham et al., 2014) demonstrated that increased perceptual discrimination difficulty is associated with positive rather than negative affinity.

**Table 5:** **Empirical evidence for hypotheses H3 (categorization ambiguity) and H4 (perceptual mismatch)**.

**Author/year**	**H3a**	**H3b**	**H3c**	**H3d**	**H3e**	**H4a**	**H4b**
Seyama and Nagayama, 2007						+	+
MacDorman et al., 2009						+	+
Looser and Wheatley, 2010	+	+	−	−			
Cheetham et al., 2011	+	+		−			
Mitchell et al., 2011						+	
Gray and Wegner, 2012						+	
Yamada et al., 2013	(+)		(+)				
Burleigh et al., 2013			(+)				−
Cheetham et al., 2013	+						
Cheetham et al., 2014	+	+	−	−	−		
Mäkäräinen et al., 2014							+
Total	4	3	2	3	1	4	4
+	4	3	0	0	0	4	3
−	0	0	2	3	1	0	1

*Conclusions: “+”: significant in favor of the hypothesis, and “−”: non-significant or significant against the hypothesis. Conclusions in parentheses have been omitted from total scores because of plausible threats to validity. Hypotheses: H3a—category identification, H3b—perceptual discrimination, H3c—categorical identification difficulty, H3d—opposite perceptual discrimination, H3e—perceptual discrimination difficulty, H4a—inconsistent human-likeness, H4b—atypicality*.

Two other studies demonstrating favorable evidence for H3c were excluded from the total count because of plausible threats to validity. One image morphing study (Yamada et al., 2013) demonstrated that the slowest identification task response times and the most negative likability evaluations coincided with each other; however, these results were excluded because the likability evaluations could plausibly have been influenced by morphing artifacts (cf. Section Naïve and Morbidity Hypotheses). Consistently, two participants in this study had reported spontaneously after the experiment that “they [had] evaluated the likability of the images based on the presence or absence of morphing noise” (ibid., 4). A more systematic evaluation would be necessary for deciding this issue, however. Another study (Study II in Burleigh et al., 2013) demonstrated that intermediate CG modifications between a goat-like and a fully humanlike face elicited the most eerie and unpleasant evaluations. This result was, however, not taken as evidence for the artificial–human categorization ambiguity (H3c) because the presence of categorization boundary was not tested explicitly. The reported positive uncanny valley finding is nevertheless important in the present context, because it could be interpreted as evidence that some human-likeness manipulations can lead to the uncanny valley. This finding was not included as additional evidence for the hypothesis H1a, however, because several other human-likeness manipulations in this study (Burleigh et al., 2013) did not lead to similar findings.

### Perceptual mismatch hypotheses

As illustrated in Table 5, the results provided good support for the perceptual mismatch hypotheses related to both inconsistent realism levels (H4a; 4 out of 4 studies) and sensitivity to atypical features (H4b; 3 out of 4 studies). Two studies (Seyama and Nagayama, 2007; MacDorman et al., 2009) using continuous human-likeness manipulations demonstrated that the most negative affinity evaluations were elicited when the mismatch between the realism of eyes and faces was the greatest (H4a) and when artificially enlarged eyes were paired with the most realistic (fully human) faces (H4b). Two other studies provided further support for H4a. One study (Mitchell et al., 2011), which had used a factorial design between the realism of a face (robot or human) and voice (synthetic or human), demonstrated that mismatched face–voice pairs elicited higher eeriness than similar matched pairs. This result was included as support for H4a, although it should be noticed that these results are somewhat limited because only one pair of stimuli were used in the study. Another study (Gray and Wegner, 2012) with conceptual stimuli demonstrated that machines with characteristically human experiences (i.e., capability to feel) and humans without such experiences were considered unnerving.

Consistently with H4b, one additional study (Mäkäräinen et al., 2014) in Table 5 demonstrated that unnaturally exaggerated facial expressions were rated as more strange on increasingly humanlike faces. Contrary to H4b, one other study (Burleigh et al., 2013) failed to demonstrate higher eeriness or unpleasantness for increasingly realistic faces. Although this non-significant finding was included in the total count, it is possible that this result could have been specific to the atypical feature (rolled-back eye) used in the study. Unlike enlarged eyes (e.g., Seyama and Nagayama, 2007), for example, such features could appear disturbing both on human and artificial faces.

Some studies that were excluded during the initial selection because they were not fully consistent with the specific formulation of the atypicality hypothesis (H4b) could nevertheless provide further evidence for it. One previous study (Green et al., 2008) demonstrated that individuals show greater agreement when judging the “best looking” facial proportions of human rather than artificial faces. Similar greater agreement for more realistic CG textures was demonstrated also in the second study of MacDorman et al. (2009). Furthermore, the third study in the same article showed that extreme facial proportions were considered the most eerie at close to humanlike levels. These results strengthen the view that individuals are more sensitive and less tolerant to deviations from typical norms when judging human faces.

## Discussion

This review considered evidence for the uncanny valley hypothesis (Mori, 2012) based on a framework of specific hypotheses motivated by previous literature. The results showed that whereas all human-likeness manipulations do not automatically lead to the uncanny valley, positive uncanny valley findings have been reported in studies using perceptually mismatching stimuli. In particular, positive uncanny valley findings have been reported for stimuli in which the realism levels of artificial and humanlike features are inconsistent with each other (e.g., human eyes on an artificial face) or in which atypical features (e.g., grossly enlarged eyes) are present on humanlike faces.

### Evidence for different kinds of uncanny valleys

Given that the original uncanny valley formulation did not provide specific guidelines for operationalizing human-likeness, we first considered the straightforward prediction that any kind of successful human-likeness manipulation would lead to the characteristic U-shaped affinity curve at almost humanlike levels. The reviewed studies, which had used various human-likeness manipulations, provided very little support for this hypothesis. Nonlinear uncanny valley effects were found only in two studies that had studied images of hands (Poliakoff et al., 2013 and a continuous CG modification between nonhuman and human faces (Study II in Burleigh et al., 2013). Whether these results could be explained by chance, some characteristics specific to these stimuli or by the other reviewed hypotheses (e.g., categorization ambiguity or perceptual mismatch) remains an open question. The absence of evidence for the naïve uncanny valley hypothesis suggests that all kinds of human-likeness manipulations do not automatically lead to the uncanny valley. This would also suggest that individual studies using only one type of human-likeness manipulation should not be taken as conclusive evidence for the existence or nonexistence of the uncanny valley.

The original uncanny valley formulation also led to the secondary prediction that any kind of human-likeness manipulations would elicit linear increases in experienced affinity. This prediction was supported by the bulk of studies. This suggests that as a general rule, increasing human-likeness is associated with more positive experiences. Exceptions to this general rule could be possible, however, given that different kinds of human-likeness manipulations were not considered systematically in the present review.

We have suggested that Mori used corpses and zombies only as metaphorical examples when discussing threat avoidance as a possible explanation for the uncanny valley. Because these examples could nevertheless be taken literally, we also considered the hypothesis that such morbid characters would elicit negative affinity. Not surprisingly, this hypothesis received support. The inclusion of this hypothesis was successful because it helped us avoid drawing false conclusions for the other hypotheses. We conclude that empirical studies should not use purposefully morbid characters to test the existence of the uncanny valley (such stimuli could, of course, be included for other purposes). Although another possible confound, purposeful aesthetic, could also have originated from a literal interpretation of the original examples, this issue did not seem to affect any of the reviewed studies.

The original uncanny valley formulation proposed that movement would amplify the characteristic uncanny curve. The reviewed studies did not support this prediction. In contrast, the reviewed studies again demonstrated a linear relationship between affinity and the human-likeness of movement patterns. Furthermore, no nonlinear uncanny valley effects were observed. This suggests that movement information imposes similar linear effects on affinity as any other variation in human-likeness. However, it should be noticed that refined uncanny valley hypotheses (see below) have up to date been studied using only static stimuli, and that movement could possibly amplify their effects.

An alternative claim to the prediction that any kind of human-likeness manipulation leads to the uncanny valley would be that the uncanny valley phenomenon is manifested only under specific conditions. For evaluating this possibility, we considered empirical evidence for two refined uncanny valley proposals as they have been presented in existing literature. First, we considered the claim that the uncanny valley would be caused by an artificial–human categorization ambiguity. Although the reviewed studies demonstrated that morphed artificial–human face continua are perceived categorically, we were able to identify only tentative evidence for negative affinity in the vicinity of category boundary. Taken together, these results suggest that the uncanny valley phenomenon could not be explained solely in terms of categorical perception. However, given the small number of reviewed studies, more conclusive results could yet be obtained in future studies. The uncanny valley hypothesis could also be interpreted such that it predicts greater perceptual discrimination difficulty and more negative affect in the vicinity of category boundary (cf. Cheetham et al., 2014). Neither of these hypotheses was supported by the reviewed evidence.

Second, we considered two different perceptual mismatch hypotheses for the uncanny valley. The first hypothesis predicted that the negative affinity associated with the uncanny valley would be caused by inconsistent realism levels (e.g., artificial eyes on a humanlike face or vice versa). The second hypothesis predicted that such negative affinity would be elicited by heightened sensitivity to atypical features (e.g., grossly enlarged eyes) on humanlike characters. Both of these hypotheses received support from the reviewed studies. This finding is important because it confirms the existence of the uncanny valley at least under some specific conditions. Although previous reviews have presented categorization difficulty and perceptual mismatch hypotheses separately (e.g., Pollick, 2010), we are not aware that a further distinction would have been made between different perceptual mismatch hypotheses. Notably, the reviewed inconsistency and atypicality hypotheses lead to slightly different symmetric and asymmetric predictions. That is, the inconsistency hypothesis would predict that both artificial features on humanlike characters and humanlike features on artificial characters will elicit negative affinity, whereas the atypicality hypothesis would predict atypicality effects only for humanlike stimuli. Because both predictions received support, this suggests that inconsistent realism levels and atypical features could represent different conditions leading to the uncanny valley.

### Open research questions

The present review raises several open questions for the uncanny valley research. One of these is the relation between the perceptual mismatch and categorization ambiguity hypotheses, which are not necessarily independent from each other. For example, it is possible that realism level inconsistency and feature atypicality effects could be reduced to categorical perception. This idea could possibly be tested by varying the level of inconsistency between features (e.g., by morphing eyes and faces separately as in Seyama and Nagayama, 2007) or by varying the level of feature atypicality (e.g., by varying the eye size of artificial and human faces), and testing whether such continua would fulfill the category identification and perceptual discrimination criteria for categorical perception (Repp, 1984; Harnad, 1987). If these criteria were fulfilled, the results would link these effects to the broader framework of categorical perception.

Another open question relates to whether any kind of perceptual mismatch would lead to the uncanny valley or whether this effect would apply the best or even exclusively to specific features. For example, it might not be a coincidence that two of the reviewed studies demonstrated a perceptual mismatch effect for inconsistent realism levels specifically between the eyes and faces and specifically for enlarged eyes presented on human faces (Seyama and Nagayama, 2007; MacDorman et al., 2009). One of the earliest reviews on the uncanny valley suggested that the eyes would have a special role in producing the uncanny valley (Brenton et al., 2005). Consistently, one image morphing study has demonstrated that human-likeness manipulations of eyes explain most (albeit not all) of the perceived animacy of faces (Looser and Wheatley, 2010). Similarly, one eye tracking study has demonstrated that eyes receive longer gaze dwell time on categorically ambiguous than on categorically unambiguous artificial faces (Cheetham et al., 2013). To our knowledge, the previous suggestion that negative affinity would be caused by inconsistent static and dynamic information (Brenton et al., 2005; Pollick, 2010) also remains unexplored.

The lack of universally agreed upon operational definition for the affinity dimension is a critical issue for uncanny valley studies. The self-report items eeriness, likability, familiarity, and affinity could be derived from Mori's (1970) original formulation. Unfortunately, an inspection of the reviewed articles (Table S1) reveals that none of these single terms alone have been adopted in more than half of the reviewed articles, even after similar terms would be considered as their synonyms (e.g., creepy and strange for eerie; pleasant or appealing for likable; and strange–familiar for familiar). Furthermore, although these items are consistent with the original formulation, they are not necessarily theoretically justified. One starting point for operationalizing affinity could be the questionnaire developed by Ho and MacDorman (2010). In the present investigation, we have defined affinity in terms of perceptual familiarity and emotional valence. However, these constructs are clearly separate from each other, and their relation in the uncanny valley context would merit further investigation.

Future studies could also consider the possible influences of image morphing artifacts on uncanny valley findings, for example by conducting independent image quality evaluations for morphed stimuli. Although the risk of image morphing artifacts can be diminished considerably by following the guidelines of Cheetham and Jäncke (2013), it is nevertheless possible that all confounding factors would not be avoided. Specifically, some ghosting for subtle facial features that are present in only one of the original images and slight blurring of contours generated by color interpolation could be unavoidable. By the nature of image morphing procedure, middle images in the series of morphed images are the most processed (in a technical sense) and hence they differ the most from natural images that constitute the endpoints of the series. Assuming that morphing artifacts were a realistic concern, the level of visual distortions produced by morphing would hence increase toward the middle of the generated human-likeness continua. The effects of such visual distortions would likely depend on the adopted research question and experimental design, however. Visual distortions, which would likely elicit negative evaluations, could lead to false negative affinity findings at the middle of the scale. On the other hand, it seems unlikely that visual distortions would explain the enhanced discrimination of stimuli straddling the scale middle (i.e., category boundary), as has been reported in typical categorical perception studies. If discrimination were based on comparing visual distortion levels, discrimination should on the contrary be enhanced for adjacent images that are located on either the left or right sides of the scale middle (i.e., for images with different distortion levels) but decreased for images that straddle the scale middle (i.e., for images with symmetric distortion levels).

### Limitations

A plausible limitation related to our conceptual analysis of the original uncanny valley formulation (Mori, 2012) is that we have relied on its English translation and other secondary sources instead of the original article written in Japanese.

Given our inclusion criteria, we have only considered studies that have operationalized affinity by self-report measures. We acknowledge that the heterogeneity of self-report items used in the previous studies has significantly reduced the value of comparing their results with one another. Another consequence is that we have omitted several relevant studies that have used physiological and behavioral measures, such as gaze tracking (e.g., Shimada et al., 2006) and haemodynamic response measurements in the brain (e.g., Chaminade et al., 2007; Saygin et al., 2012). It could also be argued that identification task response times, which have already been utilized in some categorical studies (e.g., Looser and Wheatley, 2010; Cheetham et al., 2011), would in fact be good operational definitions of perceptual familiarity. A justification for the present focus on self-report measurements is that their results are easier to interpret than those of physiological or behavioral measures. On the other hand, it should be acknowledged that physiological and behavioral measures could possibly avoid the present ambiguities related to self-report items.

The present conclusions depend on the adopted evaluation criteria, which are to some extent open to subjective interpretations. The function of these criteria was to avoid drawing false conclusions for our hypotheses; consequently, the criteria focused on plausible threats to conclusions that could be drawn from individual studies. We have attempted to facilitate the critical evaluation of this procedure by making it as transparent as possible. Because all possible aspects of experimental validity were not covered, the adopted criteria cannot and should not be taken as evidence for the experimental validity of the evaluated studies themselves. It should also be noticed that although we have specified the minimal human-likeness levels required for testing each hypothesis, this has been done solely for covering as many studies as possible. These minimal levels should hence not be taken as practical guidelines for empirical studies.

Although we have considered only the categorization ambiguity and perceptual mismatch explanations for the uncanny valley, it is worth noting that several other explanations have also been suggested (e.g., see MacDorman and Ishiguro, 2006). For example, it has been suggested that realistic appearance would elicit unrealistic cognitive expectations (expectation violation); that non-lifelike characters would trigger innate fear of death (terror management); and that some artificial characters would be eerie because they appear unfit, infertile, ill, or elicit other evolutionarily motivated aversive responses (evolutionary aesthetics). These explanations operate at different levels—the first two refer to proximate causes (i.e., how the uncanny valley is caused), whereas the evolutionary explanation refers to an ultimate cause (why the uncanny valley exists; cf. Scott-Phillips et al., 2011). Other refinements of the uncanny valley theory have suggested, for example, that behavior that is consistent with a character's appearance will lead to more positive reactions (i.e., a synergy effect; Minato et al., 2004; Ishiguro, 2006). Although these are all empirically testable hypotheses, we have not included them in the present review because they are either similar to the already included hypotheses (e.g., expectation violation vs. inconsistent realism hypotheses) or because they address higher-level topics that seem to presuppose the existence of the uncanny valley in one form or another.

We also acknowledge a recent refinement of the categorization ambiguity hypothesis, which has been suggested in two other articles of the present *Frontiers* Research Topic. As discussed by Schoenherr and Burleigh (2015), the uncanny valley could represent an overarching “inverse mere-exposure effect” (ibid., 3), in which negative affect is caused by a lack of exposure to specific stimuli or stimulus categories (e.g., the authors cite the octopus as a species that is mundanely difficult to categorize). Burleigh and Schoenherr (2015) extend this idea by demonstrating that categorization ambiguity and the frequency of exposure to specific within-category stimuli contribute independently to the uncanny valley. For example, novel stimuli that were extrapolations of their original training stimuli were categorized easily but were nevertheless considered more eerie than stimuli within their training set. These recent considerations suggest that the categorization ambiguity hypothesis alone would not necessarily be sufficient for predicting emotional responses to the uncanny valley.

### Importance and implications for research and practice

Previous articles have already reviewed the uncanny valley phenomenon (e.g., Brenton et al., 2005; Gee et al., 2005; Hanson, 2005; Ishiguro, 2007; Eberle, 2009; Pollick, 2010; Tondu and Bardou, 2011; Zlotowski et al., 2013) and explicated, for example, the categorization ambiguity (e.g., Cheetham et al., 2011) and perceptual mismatch (e.g., MacDorman et al., 2009) hypotheses. However, to our knowledge, this article is the first systematic review of the empirical evidence for the uncanny valley. Conceptual analysis of the uncanny valley and consideration of plausible threats to the conclusions drawn from previous studies to the present hypotheses were used to improve the accuracy of our conclusions. The main contribution of the present article is the conclusion that all kinds of imaginable human-likeness manipulations do not automatically lead to the uncanny valley.

The practical implications of the present findings for computer animators and human-computer or human-robot interaction developers hinge on whether these findings can be generalized to realistic stimuli and contexts—that is, whether they are externally valid (the somewhat redundant term ecological validity could also be used; cf. Kvavilashvili and Ellis, 2004). The present review failed to identify direct evidence for or against the uncanny valley in realistic stimuli, with the exception of some tentative findings (Flach et al., 2012; for other excluded but relevant studies, see Schneider et al., 2007; Tinwell, 2009; Tinwell et al., 2010). However, the reviewed results for artificial but well-controlled stimuli should be generalizable to computer animations and other realistic stimuli as well, given that the experimental stimuli clearly represented phenomena that would be likely to exist also in the real world (cf. Kvavilashvili and Ellis, 2004). For example, it is easy to imagine real computer-animated characters, whose individual features differ from each other with respect to their realism (i.e., perceptual mismatch due to inconsistent realism).

The present results could be taken to encourage the development of increasingly realistic computer animations (and other artificial characters), given that more humanlike characters were in general found to elicit more positive affinity. However, the perceptual mismatch results suggest that the uncanny valley remains a plausible threat for such characters. A generally humanlike character with subtle flaws in some focal features (e.g., eyes), would be likely to elicit negative affinity. The reviewed findings that individuals are increasingly sensitive to atypical features on more humanlike characters would suggest that avoiding the uncanny valley will become exponentially more difficult as the characters' overall appearance approaches the level of full human-likeness. This does not mean that computer animators or robotics researchers should shy away from the grand challenge of creating fully humanlike artificial entities. However, for many practical applications, there may be certain wisdom in the Mori's (1970) original advice of escaping the uncanny valley by attempting to design only moderately humanlike entities.

## Conclusion

Taken together, the present review suggested that although not any kind of human-likeness manipulation leads to the uncanny valley, the uncanny valley could be caused by more specific perceptual mismatch conditions. Such conditions could originate, at least, from inconsistent realism levels between individual features (e.g., artificial eyes on a humanlike face) or from the presence of atypical features (e.g., atypically large eyes) on an otherwise humanlike character. Categorical perception of human-likeness continua ranging from artificial to human was supported; however, the present findings failed to support the suggestion that categorization ambiguity would be associated with experienced negative affinity. The results also highlight the need for developing a unified metric for evaluating the subjective, perceptual, and emotional experiences associated with the uncanny valley.

### Conflict of interest statement

The authors declare that the research was conducted in the absence of any commercial or financial relationships that could be construed as a potential conflict of interest.
