# Supplementary material for: A review of empirical evidence on different uncanny valley hypotheses: support for perceptual mismatch as one road to the valley of eeriness
Source: Front Psychol. 2015 Apr 10;6:390. doi: 10.3389/fpsyg.2015.00390 (PMC4392592; doi:10.3389/fpsyg.2015.00390)
Supplement: Supplementary file 1 [file Table1.PDF]

Supplementary Table S1 Detailed description of selected empirical uncanny valley studies (sorted by year and authors).

Author/year [type][a]	N[b]	Stimuli [modality][c]	HL manipulat.[d]	Task[e]	HL measures[f]	AF measures[g]	Possible threats[h]
**Seyama and Nagayama 2007** [J]	see below	Faces; see below [S]	Morphing; see below	Self-report	None	Pleasant (5)	No HL check, morphing artifacts
": Study I.1	49 (20)	Doll and CG–human (3)	Morphing (11)	"	"	"	
": Study I.2	37 (15)	CG–human (1)	Morphing (21)	"	"	"	
": Study II	45 (22)	Doll–human (1)	Morphing: face and eyes separately (11)	"	"	"	
": Study III	40 (33) + 24 (19)	Doll–human (1) + CG–human (1)	Morphing and eye size separately (16)	"	"	"	
": Study IV	47 (26)	Doll–human (4)	Morphing and eye size separately (16)	"	"	"	
**MacDorman et al. 2009** [J]	see below	CG face (1) [S]	CG; see below	Self-report	see below	Reassuring-eerie and ugly-attractive (7)	Narrow stimulus set (only one CG model), narrow HL range (only CG faces)
": Study I	458 (275) + 407 (260)[j]	"	CG realism (11) * texture (3)	"	Humanlike (11)	"	
": Study IV	459 (329)	"	CG realism: face (5) * eyes (5) separately * eye size (2)	"	Artificial-natural (7)	"	
**Looser and Wheatley 2010** [J]	see below	Faces; see below [S]	see below	see below	see below	see below	
": Study I	60 + 46[j]	Statues and dolls–human (20)	Morphing (11)	Self-report and free adjustment	Animacy (7) and just-noticeable-animacy (slider)	Pleasantness (7)	
": Study II	25	Morphed pairs (8)	Category congruity (3)	Discrimination	Same-different	None	
": Study III	27	same as in Study I	same as in Study I	Self-report	Animacy (7)	None	

Author/year [type][a]	N[b]	Stimuli [modality][c]	HL manipulat.[d]	Task[e]	HL measures[f]	AF measures[g]	Possible threats[h]
**Cheetham et al. 2011** [J]	see below	Faces; see below [S]	Morphing; see below	see below	see below	see below	
": Study I	25 (13)	CG–human (32)	Morphing (13)	Identification	Artificial-human (2)	None	
": Study II	20 (9)	Morphed pairs (32)	Category congruity (3)	Discrimination	Same-different (2)	None	
**Mitchell et al. 2011** [J]	48 (28)	A/v robot-human videos (1) [M]	A/v congruity (2*2)	Self-report	Six items (7)[k]	Eight eeriness items (7), five warmth items (5)[k]	Narrow stimulus set (only one stimulus pair)
**Thompson et al. 2011** [J]	40 (18)	Animated CG characters (2) [M]	Motion distortion type (3) * level (10)	Self-report	Humanlike (7)	Familiar and eerie (7)	
**Gray and Wegner 2012** [J]	see below	see below	see below	Self-report	Two experience and agency items (5)	Uneasy, unnerved, creeped out (5)	
": Study II	45 (22)	Concept: supercomputer (1)	Computer type (normal, with-experience, with-agency)	"	"	"	
": Study III	44	Concept: person (1)	Mind type (normal, without agency, without experience)	"	"	"	
**McDonnell et al. 2012** [J]	see below	CG face; see below	see below	Self-report	see below	see below	Narrow stimulus set (only one CG face), outlier stimuli (ill character), narrow HL range (only CG faces)
": Study I	17 (7)	CG face (1) [S+M]	Rendering style (10)	"	Abstract-realistic (7)	Appealing, familiar, eerie-reassuring, friendly, trustworthy (7)	

Author/year [type][a]	N[b]	Stimuli [modality][c]	HL manipulat.[d]	Task[e]	HL measures[f]	AF measures[g]	Possible threats[h]
**Yamada et al. 2013** [J]	see below	Faces; see below [S]	Morphing (11)	Identification (HL) and self-report (AF)	Artificial-human (2), RT	Likable (7)	Morphing artifacts
": Study I	12	Cartoon–human (1)	"	"	"	"	Narrow stimulus set (only one morphed stimulus pair)
": Study II	10	Cartoon–real (1), real–stuffed (1), and real–cartoon dog (1)	"	"	"	"	
": Study III	10	Male–female (1) and male A–male B (1)	"	"	"	"	
**Burleigh et al. 2013** [J]	see below	CG faces [l]; see below [S]	CG modifications; see below	Self-report	Humanlike (7)	see below	Categorical perception not tested (H3c), narrow HL range (only CG faces)
": Study I	164 (85)[l]	CG faces (4)[m]	Realism (7) * prototypicality (7)	"	"	Eerie, fearful, disgusting, and attractive (7)	
": Study II	47 (28)	CG faces (2)[m]	Prototypicality (7) or texture color (7) * atypicality (7)	"	"	Eerie and pleasant (7)	Possible outlier stimulus (rolled-back eye)
**Carter et al. 2013** [C]	29	Animated action sequences (12) [M]	Character (4): human, CG, toon rendered CG, robotic CG	Self-report	Lifelike (7)	Common-strange, pleasant, comforting-creepy, and engaging-repulsive (7); likable (2)	
**Cheetham et al. 2013** [J]	60 (31)	CG–human faces (10)	Morphing (15)	Identification (and gaze tracking)	Artificial-human (2), RT	None	
**Poliakoff et al. 2013** [J]	43 (36)	Hands (22) [S]	Mechanical (6), prosthetic (9), and human (7)	Self-report	Humanlike (9)	Eerie (9)	Only quadratic correlation tested (i.e., H1b not tested explicitly)

Author/year [type][a]	N[b]	Stimuli [modality][c]	HL manipulat.[d]	Task[e]	HL measures[f]	AF measures[g]	Possible threats[h]
**Cheetham et al. 2014** [J]	see below	CG–human faces (8)	Morphing (11)	see below	see below	see below	
": Study I	49 (29)	"	"	Identification and discrimination	Artificial–human (2) and ABX task (2)[n]; RT	None	
": Study II	49 (34)	"	"	Identification, discrimination, and self-report	"	Familiarity (5)	
**Mäkäräinen et al. 2014** [J]	32 (22)	Facial expressions (3) [S]	Realism (6) * exaggeration (6)	Self-report	Intensity (VAS)	Strange (VAS)	No HL check, narrow stimulus range (only one actor)
**Rosenthal-von der Pütten and Krämer** 2014 [J]	151	Robots (40)[l] [S]	None	Self-report	Humanlike and mechanical (5)	Positive affect, negative affect, familiarity, intelligence (16)	Narrow HL range (only artificial characters)
**Piwek et al. 2014** [J]	20 + 20[j]	Full-body knocking animation (1) [S+M]	Character (7), motion distortion (6)	Self-report	Humanlike (7)	Acceptable (7)	Narrow HL range (only artificial characters), outlier stimuli (zombie, skeleton)

Note. Ditto (") – same as above; CG – computer graphics; HL – human-likeness; AF – affinity; VAS – visual analogue scale; A/v – audiovisual; RT – response time.

[a]This column lists authors and publication year. Article type is in square brackets: J – journal, C – conference.
[b]This column lists number of participants. Numbers in parentheses refer to female participants. Plus sign means that the study design involved two groups.
[c]This column lists descriptions of the stimuli. Numbers in parentheses refer to the number of original stimuli. Plus sign refers to stimuli that were presented to different groups. Stimulus modality is in square brackets: S – static, M – moving.
[d]This column lists human-likeness manipulations. Numbers in parentheses refer to the number of levels. Asterisk refers to factorial design.
[e]This column lists descriptions of tasks.
[f]This column lists human-likeness measures. Numbers in parentheses refer to the number of evaluation steps.
[g]This column lists affinity self-report measures. Numbers in parentheses refer to the number of evaluation steps.
[h]This column lists possible threats to the validity of conclusions from each study to the addressed hypotheses.
[i]In the ABX discrimination task, participant saw a pair of two images (A and B) followed by a second presentation (X) of either A or B, and was asked to select whether X was the same as A or B. Here A and B images were taken from the same morph continuum and were always separated by two morphing steps.
[j]Participant groups were assigned separately to affinity and human-likeness evaluation tasks.
[k]Self-report items were similar to Ho and MacDorman (2010).
[l]Stimuli were selected based on a pretest.
[m]Stimuli and/or task levels were counterbalanced between participants; see original article for details .
